# Supplementary material for: Description of a new low-cost and open-source audiometer and its validation with normal-hearing listeners: The Aupiometer
Source: PLoS One. 2024 Aug 9;19(8):e0306751. doi: 10.1371/journal.pone.0306751 (PMC11315275; doi:10.1371/journal.pone.0306751)
Supplement: Attachment — Submitted filename: Response to Reviewers.docx [file pone.0306751.s001.docx]

Dear Editor, dear Reviewers,

We thank you for all the comments which greatly helped to improve the quality of the manuscript. We have made corrections for each of these comments, which we detail below.

Journal requirements:

- We have corrected the format of the manuscript to match the style requirements.
- With the response provided (“we can proceed without any edits to figure 1”) following the request for clarification that we sent on 4/16/2024, Figure 1 is maintained. Modifications are nevertheless made to this figure following comments from Reviewers.

Additional Editor Comments:

- The title was modified accordingly.
- Yes, it would be possible to separate sensori-neural versus conductive hearing loss by performing a pure-tone audiometry measurement by bone conduction. We added mention of this possibility under discussion (l. 378, in the revised manuscript with track changes).

Reviewer #1:

- Major issues:
- Costs:
  - Indeed, it was important to add an estimate of computer development time (l. 87). However, we have not specified an associated monetary cost due to salary disparities between countries.
  - You are right, the cost of the Raspberry case and the power supply were missing and have been added (l. 83).
  - A photo of the rear view of the device has been added to Fig 1.
- Participants:
  - Yes, all sixteen participants have normal hearing, this is now clarified (l. 189).
  - We discussed the validity of the calibration for participants with hearing loss (l. 302).
- We discussed the use of an audiometric booth (l. 273).
- Minor issues:
- L. 32: correction made.
- L. 54: correction made.
- L. 58: correction made.
- Paragraph l. 63: correction made.
- L. 77: correction made.
- Zenodo repository: a link has been added (l. 102).

Reviewer #2:

- Major comments:
- L. 82: indeed, the orders of magnitude of the costs referred to different tests and did not allow a comparison between the devices. We have added an example of hardware cost to give a better point of comparison (e.g. USD$1000 for high-frequency audiometric headphones; while the cost of implementing tests depends on the manufacturers).
- The evaluation was carried out in an audiometric booth, as was mentioned in the manuscript, we have clarified this better (l. 202). In addition, to complete the evaluation, participants only had to press a button on the touch screen of both devices (see “Procedure” paragraph), i.e. with very intuitive usability. However, usability with other naive experimenters was not evaluated, but it was designed to be equivalent to standard audiometric devices with a simplified display.
- We propose uses of the device in telemedicine for example in workplaces or other public spaces where the calibration of the device could be carried out, because it is indeed necessary to be equipped. We have clarified this (l. 366).
- The raw data table has been added to the computer code (with a new DOI link; l. 102), thank you for pointing this out to us.
- Minor comments:
- The abstract has been completed with the main results (l. 32).
- The quality of Figure 1 has been improved.
- Figure 2 is the actual interface as it appears on the device. The font used on the device is simple. It was specified in the legend that the display of the questionnaire is cut off by the size of the screen, but that the user has a scroll bar (l. 100).
- The quality of Figure 4 has been improved to make the legends readable.

Sincerely,

Vincent Isnard
