# Supplementary material for: Description of a new low-cost and open-source audiometer and its validation with normal-hearing listeners: The Aupiometer
Source: PLoS One. 2024 Aug 9;19(8):e0306751. doi: 10.1371/journal.pone.0306751 (PMC11315275; doi:10.1371/journal.pone.0306751)
Supplement: Attachment — Submitted filename: Response to Reviewers.docx [file pone.0306751.s002.docx]

Dear Editor, dear Reviewers,

Thank you for this positive feedback regarding the main revisions of the manuscript. Concerning the last two points mentioned, the corrections have been made:

(1) Correction made: regarding the cost of the Raspberry itself, we had integrated it into the cost including the power supply, which could have led to confusion. In the latest version of the manuscript, we have separated the cost of the Raspberry from that of the power supply (L. 82).

(2) Correction made: we have improved the resolution of the figures as much as possible within the limits of the requested format. In particular, the dimensions of Figure 1(b) have been reduced to increase the resolution for the limited file size. We also increased the resolution of Figures 2 and 4 as much as possible. For Figure 2, note that the program design generates a simplified graphic rendering, regardless of the resolution.

Additionally, three corrections were made in the reference list: two dates were incorrect (L. 50 and 311), and a name was missing from the reference list (L. 471).

Sincerely,

Vincent Isnard
